# The COVID-19 pandemic, psychologists’ professional quality of life and mental health

**DOI:** 10.3389/fpsyg.2024.1339869

**Published:** 2024-04-25

**Authors:** Amy Kercher, Jodie Rahman, Mangor Pedersen

**Affiliations:** Department of Psychology and Neuroscience, Auckland University of Technology, Auckland, New Zealand

**Keywords:** psychologists, pandemic, compassion fatigue, professional quality of life, depression, stress

## Abstract

**Background:**

Psychologists are at known risk of work-related stress, secondary trauma, and burnout. The COVID-19 pandemic increased stress and anxiety for communities worldwide and corresponded with an increased demand for mental health services. Our study investigated the impact of COVID-19 on psychologists’ professional quality of life, psychological symptoms, and work-related stress in Aotearoa, New Zealand (NZ).

**Method:**

Ninety-nine registered psychologists were recruited via NZ professional psychology organizations, representing 3% of the total workforce. Survey data collected included symptoms of compassion fatigue and satisfaction, psychological symptoms, COVID-19-related stress and resilience, and professional and personal circumstances during the third year of the pandemic, 2022.

**Results:**

Seventy percent reported that their work stress had increased, and 60% reported that their caseload intensity had increased during the COVID-19 pandemic. Psychologists reported receiving little to no additional personal or professional support, while 55% reported increased personal responsibilities during the pandemic (for example, closed childcare and schools during lockdowns). High rates of compassion fatigue (burnout and secondary traumatic stress) and low resilience were reported. We observed that psychological distress was higher than the community averages before the pandemic and comparable with frontline healthcare professionals. Compassion fatigue was associated with COVID-related stress, psychological distress, years in practice, and more frequent supervision, but not with working with at-risk clients, levels of personal support, or having children at home. Despite these difficulties, high Compassion Satisfaction scores were also reported, with over 90% indicating they had no intention of leaving the profession in the foreseeable future.

**Conclusion:**

Psychologists’ compassion fatigue appears to have worsened during the COVID-19 pandemic, as have their symptoms of psychological distress. Increased workplace and clinical demands, telehealth difficulties, stress relating to the pandemic, inadequate support, and increased personal responsibilities were reported by psychologists. Mental health workforces are not immune to the personal and professional impacts of crises and are at risk of burnout and secondary traumatic stress. We hope that increased awareness and understanding of psychologists’ own difficulties during COVID-19 can be used to better tackle future crises and support mental health professionals.

## Introduction

During the pandemic, mental health service demand increases were reported worldwide (Saha et al., 2020; Benton et al., 2022; Deng et al., 2023; Sicouri et al., 2023), specifically in Aotearoa, New Zealand (NZ; Every-Palmer et al., 2020; Freeman et al., 2021; Gasteiger et al., 2021; Officer et al., 2022). Healthcare professionals (HCPs) faced the unusual challenge of sharing stressful situations with their patients, as navigating uncertain health risks, lockdowns, travel restrictions, and financial disruptions coincided with increased client distress and severity. In NZ, government-mandated restrictions were among the strictest and most effective in reducing the spread and mortality of COVID-19, such that widespread community transmission was not seen until the 2022 Omicron outbreak. At the time of this study in 2022, extended lockdowns had only recently ended, vaccination rates were high, and community transmission was increasing (Ministry of Health, 2022). Many psychologists continued to work remotely via telehealth.

The impact of the pandemic on HCPs has been reported by frontline medical workers, with rates of stress and anxiety particularly noted (Buselli et al., 2020; Bell et al., 2021). However, little research has been conducted on mental health professionals. During the pandemic, psychologists reported clients needing care as much or more than before, as the stress of the circumstances compounded existing challenges (British Psychological Society, 2020). Unprecedented numbers of people attended NZ hospitals for mental health emergencies, particularly among young people (Every-Palmer et al., 2020; Freeman et al., 2021; Gasteiger et al., 2021; Officer et al., 2022). However, the effects on psychologists went largely unexamined, with little consideration given to these professionals working to support their communities.

Pre-pandemic research established a significant risk of work-related stress, vicarious trauma, and burnout symptoms among psychologists. Estimates were that between 20 and 67% of psychologists experienced symptoms of burnout (Morse et al., 2012; McCormack et al., 2018; O’Connor et al., 2018; Simpson et al., 2019). In 2021, the second year of the COVID-19 pandemic, NZ psychologists reported significantly higher rates of burnout and secondary traumatic stress than caring professionals internationally and in earlier NZ-based studies (Kercher and Gossage, 2023). Psychologists also reported difficulties, mainly working with high-risk clients, stress, and depression symptoms, which were linked with compassion fatigue. However, the specific and prolonged effects of the pandemic were not clear, which was the motivation for the current study.

During the pandemic, psychologists and other mental health professionals saw the challenge of therapy moving online, a platform many had rarely used, and which brought ethical, legal, and technical difficulties (British Psychological Society, 2020). The benefits of ongoing connections and support for clients were numerous, with clients accessing services from home. Clinical practices have since changed, with services and training programs focusing more on online delivery than ever. Anecdotally, psychologists, like many others, reported challenges from juggling remote work with children schooling at home and other caring responsibilities, but this was not being measured formally. The current study sought to assess the challenges faced by psychologists during the pandemic and developed a questionnaire for this purpose (Rahman et al., 2024).

The current study focused on the effects of COVID-19 on psychologists in NZ. Psychologists undergo prescribed training and practice under comprehensive codes of conduct and ethics and are thus a relatively homogenous and standardized sample of mental health practitioners. By the third year of the pandemic, themes were beginning to emerge in anecdotal discussions among psychologists—telehealth fatigue, personal demands, client changes, and systemic challenges. This research investigated these factors and sought to understand the effects of COVID-related stressors on psychologists’ psychological symptoms and levels of compassion fatigue.

## Method

### Participants

Online surveys were conducted with 110 registered psychologists in NZ. Of these, 99 completed the study, with 11 largely incomplete responses excluded. This represented approximately 3% of NZ’s registered psychologists (New Zealand Psychologists Board, 2021). Similar to the profession’s demographic makeup, 82% were identified as Pākeha (of European descent), 1% as Māori, 1% as Pasifika, 5% as Asian, and 11% from other backgrounds, with 92% female, 8% male, and no non-binary respondents, and a median age range of 41–45 years. The majority (84%) were married or in *de facto* relationships, 48% reported no children under 18 living in their home, 41% had one to two children, and 11% had three or more. Nine percent reported additional caregiving responsibilities (e.g., relatives with disabilities or illnesses). More than half (55%) received little to no support with personal commitments, while 23% reported adequate support and 22% good support. Approximately one-third (33%) reported additional personal stressors during the survey (e.g., health problems, housing or financial hardships, and domestic violence).

Professional characteristics varied, as shown in Appendix A. Participants reported an average of 11–15 years in practice, with 90% receiving the required monthly supervision. The NZ Psychologists Board Guidelines on Supervision recommend regular sessions, wherein discussions with a respected colleague include self-reflection, professional issues, and feedback on all elements of practice, with a focus on the quality of service, improving practice, and managing the impacts of professional work upon the supervisee (New Zealand Psychologists Board, 2021). Approximately half worked in publicly funded roles in health or hospital settings, and nearly half in private practice. Psychologists worked with varied client groups, including clients at risk of self-harm and suicide. The majority reported no intention to leave soon, with over 90% intending to remain in practice for more than 5 years.

### Measures

#### Professional quality of life scale (ProQOL)

The Professional Quality of Life Scale (ProQOL; Stamm, 2010) is a widely used measure of the positive and negative aspects of mental health work, comprising three subscales. Thirty items are answered on a Likert Scale (from 1 = never to 5 = very often). Compassion satisfaction (CS) represents the feeling of satisfaction and reward derived from one’s work, a positive outcome. Burnout (BO) incorporates feelings of disconnection, hopelessness, and ineffectiveness in one’s role, while Secondary Traumatic Stress (STS) assesses vicarious trauma symptoms, including fear and overwhelm. These two negative outcomes are summed to represent compassion fatigue (CF), the negative impact of caring work (Stamm, 2002, 2010; Larsen and Stamm, 2008). Given the high level of collinearity between these two subscales, this composite negative outcome was used in multivariate analyses to allow the exploration of other variables. At the same time, BO and STS are considered compared to previous studies that reported these separate constructs.

The ProQOL has strong psychometric properties, with each subscale showing good construct validity and internal consistency (α from 0.75 to 0.88, Stamm, 2010). In the current study, Cronbach’s alpha was 0.77 for BO, 0.83 for STS, and 0.89 for CS.

#### Depression, anxiety, stress scale (DASS-21)

Symptoms of psychological distress were measured using the DASS-21, a commonly used questionnaire (Lovibond and Lovibond, 1995). Twenty-one items are answered on a four-point Likert Scale (0 to 3), with seven items assessing symptoms of depression, anxiety, and stress, respectively. Scores are doubled to allow comparisons with the original DASS-42 instrument (Crawford et al., 2011). The DASS-21 is widely recognized for its robust psychometric properties (Medvedev et al., 2020). In the current study, Cronbach’s alpha was 0.85 (depression), 0.77 (anxiety), and 0.76 (stress). Overall symptoms of psychological distress can be measured from the total DASS-21 symptom score (Alfonsson et al., 2017; Zanon et al., 2021), which was used here in multivariate analyses to allow consideration of other variables without the multicollinearity between DASS-21 subscales.

#### Connor–Davidson resilience scale (CD-RISC-10)

The 10-item Connor–Davidson Resilience Scale (CD-RISC-10) measures resilience (Connor and Davidson, 2003; Davidson, 2018). A Likert scale (0 = not true at all, 4 = true nearly all the time) assesses stress coping abilities, with a final score of the sum of responses (0–40) and higher scores indicating higher resilience. The CD-RISC-10 has good psychometric properties (Campbell-Sills et al., 2009; Davidson, 2018), with Cronbach’s alpha here of 0.80.

#### COVID-19 related stress (CVRS)

The CVRS was developed for the current study specifically to assess the negative impact of the COVID-19 pandemic on those working in the mental healthcare sector. Five questions were based on the findings of a qualitative report by the British Psychological Society (2020), which reported experiences of the pandemic among psychologists, with two additional questions created by the authors. The measure presents seven statements on a five-point Likert scale (1 = not true at all, 5 = true nearly all the time; see Appendix B). A higher score indicates a greater level of COVID-19-related stress. The reliability of this scale was established by Rahman et al. (2024), with Cronbach’s alpha of 0.83 indicating high internal consistency. In addition, the Kaiser–Meyer–Olkin (KMO) tests and Bartlett’s sphericity tests were supported using exploratory factor analysis (EFA), which confirmed a one-factor solution.

#### Survey questions

The questionnaire also surveyed psychologists’ professional and personal circumstances, including types of work and client presentations, frequency of work with at-risk clients (from 0 = ‘never’ to 3 = ‘very often’), therapeutic practices, supervision and professional support, demographics, family and caring responsibilities, and personal support.

### Procedure

Participants were recruited via social media and email invitations shared by the New Zealand Psychological Society and New Zealand College of Clinical Psychologists. Almost all psychologists in NZ are members of one of these organizations. Participants gave informed consent and completed the survey via the online research platform Qualtrics, with no identifying information recorded. Ethical approval was granted by the Auckland University of Technology Ethics Committee (21/54, 8th April 2022).

### Data analysis

Analyses were conducted using the software package Jamovi (v.1.6.23) and online t-test calculators.^1^ Reliability analyses were conducted for all scales before analyses (as above). The properties of the CVRS were investigated through exploratory factor analysis (EFA) using principal axis factoring extraction and Obliman rotation (see Appendix C). Based on eigenvalues of more than one, the unidimensional one-factor solution was justified with all factor loadings larger than 0.40.

Independent t-tests were performed to compare our sample with previous studies of psychologists and health professionals. Welch’s t-tests were used where the variances were unequal (Delacre et al., 2017). Spearman’s rho correlations were calculated to investigate the associations between the key variables. Multicollinearity was investigated due to the correlation between variables. However, the variance inflation factors (VIFs) were less than 5, suggesting that the multicollinearity is not strong enough to prevent a multiple linear regression (MLR). Bivariate correlations were conducted. An MLR was then conducted to investigate the impact of CVRS, distress, and workplace characteristics on CF.

## Results

### Descriptive statistics and comparisons

#### Distress and compassion fatigue

Descriptive statistics for the distress (DASS-21), resilience (CD-RISC), COVID-19-related stress (CVRS) and burnout, secondary traumatic stress, and compassion satisfaction (ProQOL) scores are presented in Table 1.

**Table 1:** Descriptive statistics for the current sample (*N* = 99) and comparisons with previous studies.

Variables	*M*	SD	Range	*T*-test comparison with previous norms
DASS -21 depression	7.5	6.1	0–38	*N* = 785, *M* = 5.0, SD = 7.5; *t*(138) = 3.7, *p* < .001^a^ *N* = 3,770, *M* = 7.8, SD = 8.3; *t*(107) = 0.48, *p* > .05^b^
DASS -21 anxiety	4.8	5.6	0–18	*N* = 785, *M* = 3.4, SD = 5.1; *t*(119) = 2.27, *p* < .05^a^ *N* = 3,770, *M* = 6.8, SD = 7.5; *t*(107) = 3.5, *p* < .001^b^
DASS -21 stress	13.3	6.6	0–40	*N* = 785, *M* = 8.10, SD = 8.40; *t*(141) = 7.1, *p* < .0001^a^ *N* = 3,770, *M =* 14.0, SD = 9.6; *t*(109) = 1.0, *p* > .05^b^
ProQOL BO	24.6	5.1	13–36	*N* = 224, M = 20.78 SD = 5.16; *t*(189) = 6.2, *p* < .0001^c^ *N* = 265, M = 19.8, SD = 5.0; *t*(172) = 8.0, *p* < .0001^d^
ProQOL STS	20.7	5.6	12–43	*N* = 224, *M* = 16.34, SD = 3.61; *t*(135) = 7.2, *p* < .0001^c^ *N* = 265, M = 18.0, SD = 5.6; *t*(362) = 4.1, *p* < .0001^d^
ProQOL CS	37.6	5.8	25–49	*N* = 224, *M* = 38.6, SD = 11.9; *t*(318) = 1.0, *p* > .05^c^ *N* = 265, *M* = 38.2, SD = 7.0; *t*(210) = 0.4, *p* > .05^d^
CD-RISC resilience	27.9	4.1	15–28	*N* = 224, *M* = 30.0 SD = 4.8; *t*(215) = 4.0, *p* < .0001^c^ *N* = 458, *M* = 32.1, SD = 5.8; *t*(193) = 8.5, *p* < .0001^e^

a Compared with a community sample before the pandemic (Crawford et al., 2011).

b Compared with a frontline hospital staff during COVID-19 in Australia (Hammond et al., 2021).

c Compared with a previous sample of NZ psychologists (McCormick, 2014).

d Compared with a sample of frontline hospital staff during the pandemic in Italy (Buselli et al., 2020).

e Compared with a US community sample (Davidson, 2018).

Independent sample t-tests compared our sample with previous norms (see Table 1). Notably, NZ psychologists reported significantly higher average depression, anxiety, and stress than pre-pandemic community norms and higher average burnout and secondary traumatic stress than pre-pandemic psychologists. Distress averages were comparable to those reported by frontline HCPs during the pandemic’s peak, except for anxiety, which was higher for medical personnel. Resilience was lower than pre-pandemic psychologists and community norms. Compassion satisfaction was comparable with previous samples of psychologists and frontline HCPs.

#### Effects of the COVID-19 pandemic

**Figure 1:**
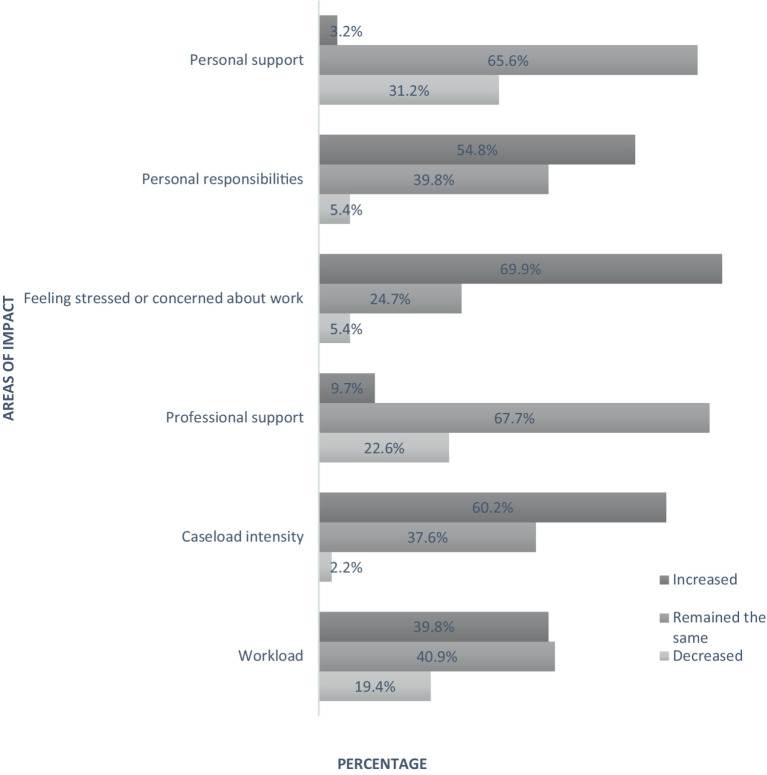
Effects of COVID-19 lockdowns and restrictions on New Zealand psychologists (*N* = 99).

Using our new COVID-19-related stress (CVRS) scale measure, psychologists reported a range of scores from 7 to 33 (*M* = 19.2, SD = 5.5). During the pandemic, most psychologists also reported increased stress or concern about their work (69.9%), increased caseload intensity (60.2%), and increased personal responsibilities (54.8%); however, nearly all reported no increase in professional support (90.3% the same or decreased) or personal support (97% the same or decreased; see Figure 1).

### Bivariate analyses

Due to collinearity between the subscales, the composite Compassion Fatigue and total DASS-21 distress scores were used in multivariate analyses. Correlations were considered based on the hypotheses, with expected relationships between stressors (children at home, low support in the workplace or home, low experience, and more frequent work with at-risk clients) and experiences of distress (CVRS, DASS-21, CF). In contrast, supervision and high support were expected to be protective, and resilience and CS would show the opposite effects (see Table 2). These hypotheses were supported—CF is strongly associated with psychological distress, and both are strongly related to CVRS. Having more children at home was associated with increased COVID-related stress. Interestingly, CF positively correlates with increased supervision but not with years in practice. Having more experience appears slightly protective against distress. Those working with at-risk clients report higher resilience and supervision but not higher CF or distress. No significant relationships were found for other client presentation types (mental health severity, trauma, child/adolescent, Māori, and Pasifika, *p* > 0.05).

**Table 2:** Bivariate correlations (*N* = 99).

	Compassion fatigue	DASS total	CVRS	Years in practice	Children at home	Personal support	At risk clients	Supervision	Workplace support	Compassion satisfaction
DASS Total	0.52**	–								
COVID-related stress (CVRS)	0.48**	0.54**	–							
Years in practice	0.10	−0.22*	−0.09	–						
Children at home	.0.03	0.06	0.26*	0.18	–					
Personal support	−0.06	−0.12	−0.12	0.08	0.12	–				
At risk clients	0.05	0.01	−0.10	−0.03	−0.10	−0.07	–			
Supervision	0.27**	0.09	0.07	−0.06	−0.09	0.04	0.31**	–		
Workplace support	−0.18	0.03	−0.14	−0.14	0.20	0.32*	−0.18	−0.05	–	
Compassion satisfaction	−0.50**	−0.18	−0.26*	0.03	0.14	0.15	0.00	−0.16	0.31*	–
Resilience	−0.18	−0.16	−0.03	−0.06	−0.02	−0.01	0.22*	−0.08	0.04	0.37**

** *p* < 0.01, * *p* < 0.05.

There are clear relationships between indicators of distress, stress, and CF, which were further explored in multivariate analyses. One-way ANOVA was conducted to investigate differences between public sector and private practice psychologists. However, differences in CVRS, CF, and DASS-21 total were not significant (*p* > 0.05).

### Multivariate analyses

#### Predictors of compassion fatigue

Multiple linear regression analyses were conducted to investigate the predictors of CF, considering the role of COVID-related stress and other factors hypothesized to be related. This model was significant, with 45% of the variance in CF explained by the variables listed in Table 3 (*F*(7, 82) = 9.75, *p* < 0.001. *R*^2^ = 0.45). Higher levels of psychological distress (DASS-21 total symptoms), COVID-related stress (CVRS), supervision frequency, and years in practice all predicted increases in compassion fatigue, measured during the pandemic. Having children at home, providing personal support, and working with at-risk clients were not significant predictors of CF.

**Table 3:** Multiple linear regression results: predictors of compassion fatigue (*N* = 99).

Predictor	B	SE	95% CI	*β*	*t*	*p*	Tolerance	VIF
(Intercept)	20.85	4.93	11.0–30.7		4.23	<0.001		
DASS total	0.32	0.06	0.2–0.4	0.50	5.14	<0.001	0.69	1.43
CVRS	0.336	0.17	0.0–0.7	0.21	2.11	0.04	0.67	1.49
Years in practice	1.32	0.59	0.2–2.5	0.19	2.25	0.03	0.89	1.11
Children	−0.55	0.73	−2.0–0.9	−0.07	−0.74	0.46	0.87	1.16
Personal support	−0.18	0.52	−1.2–0.9	−0.03	−0.34	0.73	0.90	1.11
At risk clients^a^	0.71	1.09	−1.4–2.9	0.06	0.68	0.50	0.85	1.18
Supervision	3.61	1.42	0.8–6.4	0.22	2.55	<0.05	0.87	1.15

a 1 = never, 2 = occasionally, 3 = fairly often, 4 = very often.

## Discussion

Psychologists in Aotearoa, NZ, reported high rates of psychological distress, burnout, and secondary traumatic stress symptoms during the third year of the pandemic (2022). While a great deal of attention has rightly focused on frontline medical workers’ wellbeing and the risk of burnout during the pandemic, our study shows that psychologists have been experiencing the same difficulties.

Psychologists had higher average scores for depression, anxiety, and stress than pre-pandemic community norms (Crawford et al., 2011) and significantly higher average burnout and secondary traumatic stress than pre-pandemic psychologists in NZ (McCormick, 2014). Notably, NZ psychologists’ average distress was comparable with symptoms reported by frontline HCPs during the peak of the pandemic in Australia (Hammond et al., 2021), except for anxiety, which was higher for medical personnel—likely due to the intensity of emergency room work during this time which would drive autonomic nervous system arousal, as measured by the DASS-21 anxiety scale.

Psychologists in NZ reported higher average burnout and secondary traumatic stress than Italian frontline healthcare professionals during the peak of the pandemic, in intensely stressful and distressing conditions (Buselli et al., 2020). This may be because burnout is a slow-onset phenomenon, culminating in work-related issues over a long period (Stamm, 2002), whereas the Italian medical community was experiencing more acute and intensive stress. However, secondary traumatic stress can have a sudden onset, so, it is surprising that our rates were higher than the Italian HCPs on this scale.

Resilience was lower than pre-pandemic NZ psychologists (McCormick, 2014) and general community norms (Davidson, 2018). The latter is particularly surprising, as mental healthcare professionals typically show high rates of resilience (Davidson, 2018) and have a professional understanding of coping strategies. Interestingly, psychologists working with more at-risk clients reported higher resilience, though this was not significantly related to compassion fatigue or other measures of distress. It could be that psychologists self-select their work, and those with higher resilience elect to work with higher-risk presentations. On the other hand, compassion satisfaction scores were comparable with previous samples of NZ psychologists and frontline HCPs, suggesting that psychologists enjoy their work and find it rewarding. These results were comparable with our earlier sample of NZ psychologists in 2021 (Kercher and Gossage, 2023), so measurement errors are unlikely—psychologists have repeatedly reported elevated distress. Similar difficulties were reported for frontline HCPs (Bell et al., 2021) and psychiatrists in NZ during the pandemic (Chambers and Frampton, 2022), but with different measures preventing direct comparisons.

The current study sought to understand the role of COVID-19-related stress, among other workplace and personal factors, in contributing to the reported levels of compassion fatigue among NZ psychologists. We found that COVID-19-related stress was a predictor of CF, over and above psychological distress, years in practice, supervision, and non-significant predictors, including personal support, having children at home, and working with at-risk clients. Interestingly, receiving more supervision was associated with increased CF—perhaps those at risk of CF are actively seeking more support or working in settings where this is offered. However, working in public or private settings was not significantly associated with CF. Those with more years of practice experience reported higher CF in a model containing the other predictors. This is unusual—often, there is a “survivorship effect” seen in burnout, where those prone to experience it leave their roles early in their careers, and those with more years of experience appear more resilient to burnout (Rupert and Morgan, 2005).

Psychologists reported increases in stress and concern about work, caseload intensity, and personal responsibilities during the pandemic, with more than 90% reporting no increases in personal or professional support. While the pandemic has since eased, with the government of Aotearoa, NZ, removing the last of the public health orders (Government NZ, 2023), ongoing pressures continue for the mental health sector. Frequent reports emphasize shortages of psychologists (Psychology Workforce Task Group, 2016; Skirrow, 2021), psychiatrists (Thabrew et al., 2017), increases in demand (Every-Palmer et al., 2022), difficulties with access (Officer et al., 2022), and waitlists (Cardwell, 2021). Arguably, this is causing a worsening cycle of severity in the community—services triage patients and see the most severe cases first, leaving those with mild-to-moderate concerns without help. Without intervention, many psychological conditions worsen over time (Ghio et al., 2015) and are associated with an increased risk of suicide (Maslow et al., 2015). Clients receive treatment when their symptoms worsen (Blayney and Kercher, 2023). As a result, psychologists report increasing severity, intensity, and concern about their work, although this was not directly associated with CF in the current study.

Clearly, the mental health sector requires increased funding and resourcing ahead of future crises. The challenges of the pandemic exacerbated workforce shortages and increased demand on a sector already under strain and the psychologists working to support their communities. Given the increasing frequency of natural disasters and other challenges in Aotearoa, NZ, and worldwide, the mental health sector needs to be better prepared for such difficulties in the future. Learning from the impact of the pandemic on psychologists, we need to focus on better supporting our health and support services and improve the resilience of mental health systems in the future.

The cross-sectional nature of our survey was a limitation of this study. Due to anonymity, we could not compare responses with the survey conducted in 2021. Still, we can track average rates of distress and professional quality of life, which were comparable across the two samples. Although our sample size (*N* = 99) was modest, we recruited only registered, practicing psychologists who undergo extensive training and practice under standardized codes of conduct and practice in Aotearoa, New Zealand, thus providing a homogenous sample. However, self-selection bias is possible, whereby those under most stress or most at risk of CF may not take the time to answer a survey. The survey was also limited in socio-cultural diversity, with most respondents from European NZ backgrounds identifying as female participants. Invitations were extended to target Māori and Pasifika psychologists’ groups; however, response rates were low. It will be important for future research to engage better tangata whenua psychologists, who are at known risk of burnout (Levy, 2002; Hemopo, 2004).

Encouragingly, our respondents reported good average rates of compassion satisfaction. Additionally, more than 90% reported that they had no intention to leave the profession soon (in contrast with NZ psychiatrists, nearly half of whom reported intention to leave, Chambers et al., 2022). Psychologists report finding their work rewarding and satisfying, reflected in the reported sense of purpose and reward both here and internationally during the pandemic (British Psychological Society, 2020).

The strong implication of this study is that psychologists face significant challenges in their roles. Combined with workforce and health system data indicating continual increases in demand and insufficient resources, it is vital that the shortage of psychologists is addressed with increased training and that the mental healthcare sector in Aotearoa, NZ, receives increased resources. While supervision and workplace support were not protective against CF here, almost all respondents received the required minimum of monthly supervision sessions—arguably, without this, distress could be even worse. Relatively few psychologists received more supervision than this despite guidelines recommending additional sessions for less experienced practitioners, new areas of work, or client crises. Supervision and professional development are generally protective against burnout and distress for mental health professionals (Yang and Hayes, 2020), while supportive workplaces and manageable demands are also critical (Maslach and Leiter, 2016). A larger sample of psychologists would allow better investigation of these potential protective factors, wherein a focus on different types of resilience and the potential role of supervision is suggested. Workplaces, the healthcare sector, and psychologists’ organizations should consider screening psychologists for burnout and secondary traumatic stress and address both demands and support within their roles.

## Conclusion

For 2 years in a row, psychologists in Aotearoa, NZ, have reported high average scores of burnout and secondary traumatic stress, as well as psychological distress and low resilience. The current study found that COVID-related stress was predictive of compassion fatigue, over and above the additional effects of psychological distress (depression, anxiety, and stress symptoms), years in practice, and supervision. Supervision, workplace support, and years in practice were not protective, and personal factors did not contribute to the risk of CF over and above the impact of COVID-related stress. In the future, it is important to assess the ongoing risk of burnout, secondary traumatic stress, and psychological distress among psychologists. Given the ongoing increases in mental health demand worldwide and the impact of the pandemic on psychologists, priority should be given to increasing resources in mental health sectors and better supporting our caring professionals.

## Data availability statement

The raw data supporting the conclusions of this article will be made available by the authors, without undue reservation.

## Ethics statement

The studies involving humans were approved by Auckland University of Technology Ethics Committee. The studies were conducted in accordance with the local legislation and institutional requirements. The participants provided informed consent to participate in this study.

## Author contributions

AK: Conceptualization, Data curation, Formal analysis, Investigation, Methodology, Project administration, Supervision, Writing – original draft, Writing – review & editing. JR: Data curation, Investigation, Project administration, Writing – original draft. MP: Formal analysis, Writing – review & editing.

## Footnotes

^1^ www.graphpad.com
