# Supplementary material for: The COVID-19 pandemic, psychologists’ professional quality of life and mental health
Source: Front Psychol. 2024 Apr 25;15:1339869. doi: 10.3389/fpsyg.2024.1339869 (PMC11079217; doi:10.3389/fpsyg.2024.1339869)
Supplement: Supplementary file 1 [file Data_Sheet_1.docx]

**Appendix A**

*Participant Characteristics*

| Professional scope | 62% clinical  17% general  13% educational  8% other |
| --- | --- |
| Years of experience | 20.6% 0-5 years  19.6% 6-10 years  23.7% 11-15 years  16.5% 16-20 years  19.6% 20 years or more |
| Workplaces | 50% government/ public sector  45% private practice  5% other (e.g. NGO, school) |
| Supervision | 48% monthly  42% fortnightly  4% weekly  6% missing |
| Client presentations (participants could indicate more than one response) | 37% severe mental health difficulties  73% moderate mental health difficulties  54% mild mental health difficulties  68% trauma  55% self-harm  54% at risk of suicide  50% children/adolescents  31% Māori clients  21% Pasifika clients |
| Intention to leave | 1.1% 0-1 years (soon)  6.4% 2-5 years  16.0% 6-10 years  14.9% 11-15 years  61.7% Upon retirement |

**Appendix B**

*The COVID-Related Stress Scale (CVRS)*

Which statement best describes your experience during the pandemic?

1. I have struggled to focus on work while dealing with personal sickness, bereavement, or stress and worry caused by the pandemic.
2. The lack of physical separation between work and home has made maintaining boundaries between professional and personal life difficult.
3. The lack of work-home separation has negatively impacted my family relationships.
4. The loss of informal support and face-to-face contact with colleagues has resulted in feelings of isolation, loss of confidence and/or motivation.
5. The prolonged impact of the pandemic on my usual ways of working has left me physically fatigued, and feeling emotionally drained and exhausted.
6. The lack of access to some of my self-care or stress-relieving activities has increased my feelings of stress. (For example, being unable to attend the gym, social events or other activities, or take breaks from family responsibilities, due to lockdown or pandemic restrictions.)
7. Have you increased any less healthy habits during the pandemic, for example increased alcohol, recreational drug use or binge eating?

*Note.* Likert scale scoring: Not true at all = 1, rarely true = 2, sometimes true = 3, often true = 4, true nearly all the time = 5.

**Appendix C**

*Results of Exploratory Factor Analysis of the CVRS*

|  | Factor  1 | Uniqueness |
| --- | --- | --- |
| I have struggled to focus on work while dealing with personal sickness, bereavement, or stress and worry caused by the pandemic. | 0.600 | 0.639 |
| The lack of physical separation between work and home has made maintaining boundaries between professional and personal life difficult. | 0.662 | 0.562 |
| The lack of work-home separation has negatively impacted my family relationships. | 0.657 | 0.569 |
| The loss of informal support and face-to-face contact with colleagues has resulted in feelings of isolation, loss of confidence and/or motivation. | 0.538 | 0.711 |
| The prolonged impact of the pandemic on my usual ways of working has left me physically fatigued, and feeling emotionally drained and exhausted. | 0.734 | 0.462 |
| The lack of access to some of my self-care or stress-relieving activities has increased my feelings of stress. | 0.739 | 0.454 |
| I increased any less healthy habits during the pandemic, for example increased alcohol, recreational drug use or binge eating. | 0.564 | 0.682 |

*Note.* ‘Minimum residual’ extraction method was used, in combination with an ‘oblimin’ rotation.
